# Oxidative Stress, Plant Natural Antioxidants, and Obesity

**DOI:** 10.3390/ijms22041786

**Published:** 2021-02-11

**Authors:** Israel Pérez-Torres, Vicente Castrejón-Téllez, María Elena Soto, María Esther Rubio-Ruiz, Linaloe Manzano-Pech, Verónica Guarner-Lans

**Affiliations:** 1 Department of Molecular Biomedicine, Instituto Nacional de Cardiología “Ignacio Chavez”, Tlalpan 14080, Mexico; israel.perez@cardiologia.org.mx (I.P.-T.); loe_mana@hotmail.com (L.M.-P.); 2 Department of Physiology, Instituto Nacional de Cardiología “Ignacio Chavez”, Tlalpan 14080, Mexico; vicente.castrejon@cardiologia.org.mx (V.C.-T.); esther.rubio@cardiologia.org.mx (M.E.R.-R.); 3 Department of Immunology, Instituto Nacional de Cardiología “Ignacio Chavez”, Tlalpan 14080, Mexico; elena.soto@cardiologia.org.mx

**Keywords:** oxidative stress, obesity, antioxidants, natural products

## Abstract

Oxidative stress is important in the pathophysiology of obesity, altering regulatory factors of mitochondrial activity, modifying the concentration of inflammation mediators associated with a large number and size of adipocytes, promoting lipogenesis, stimulating differentiation of preadipocytes to mature adipocytes, and regulating the energy balance in hypothalamic neurons that control appetite. This review discusses the participation of oxidative stress in obesity and the important groups of compounds found in plants with antioxidant properties, which include (a) polyphenols such as phenolic acids, stilbenes, flavonoids (flavonols, flavanols, anthocyanins, flavanones, flavones, flavanonols, and isoflavones), and curcuminoids (b) carotenoids, (c) capsaicinoids and casinoids, (d) isothiocyanates, (e) catechins, and (f) vitamins. Examples are analyzed, such as resveratrol, quercetin, curcumin, ferulic acid, phloretin, green tea, Hibiscus Sabdariffa, and garlic. The antioxidant activities of these compounds depend on their activities as reactive oxygen species (ROS) scavengers and on their capacity to prevent the activation of NF-κB (nuclear factor κ-light-chain-enhancer of activated B cells), and reduce the expression of target genes, including those participating in inflammation. We conclude that natural compounds have therapeutic potential for diseases mediated by oxidative stress, particularly obesity. Controlled and well-designed clinical trials are still necessary to better know the effects of these compounds.

## 1. Introduction

Obesity is a complex disease having an important public health impact worldwide, and its prevalence is increasing [1]. It is the result of an individual complex interaction of factors, including genetic predisposition, diet, metabolism, and physical activity [1]. It is related to several severe complications, such as metabolic syndrome, type 2 diabetes mellitus, cardiovascular dysfunction (CVD), and hypertension [1]. Reactive oxygen species (ROS) are a by-product of metabolism, and they play an important role in the development of obesity and its metabolic complications [2]. ROS participate as regulatory factors of mitochondrial activity; they modify the concentration of molecules taking part in inflammation, which is associated with a large number and size of adipocytes, they promote adipogenesis and lipogenesis, they stimulate the differentiation of preadipocytes to mature adipocytes, and they play an important role as agents that regulate the energy balance in hypothalamic neurons that control appetite [2,3].

Despite a variety of surgical and pharmacotherapeutic measures, there are still no risk-free and efficient weight management treatments. Lifestyle modification, changes in diet, and reduced sedentarism are currently considered as the best alternative. Phytotherapy is the targeting of health problems by the employment of plant-derived medications. Some plant extracts act as anti-obesity agents. Moreover, natural plant supplementation causes important weight loss and improves health through the neutralization of ROS. The products of plants are an alternative for the management of weight, since they constitute a source of many active chemicals, including polyphenols, flavonoids, carotenoids, catechins, isoflavones, capsaicinoids and capsinoids, such as resveratrol, quercetin, curcumin, ferulic acid, and phloretin. Some plants that have been tested for their effects on obesity are Hibiscus sabdariffa extracts, green tea, and garlic, among others. Plant-derived chemicals may improve the condition of adipose tissue in obese individuals by reducing intracellular oxidative stress (OS) [4]. In this paper, we describe the roles of ROS in obesity and the possible impact of natural antioxidants in the treatment of this disease.

## 2. Redox Balance in Obesity

**Figure 1:**
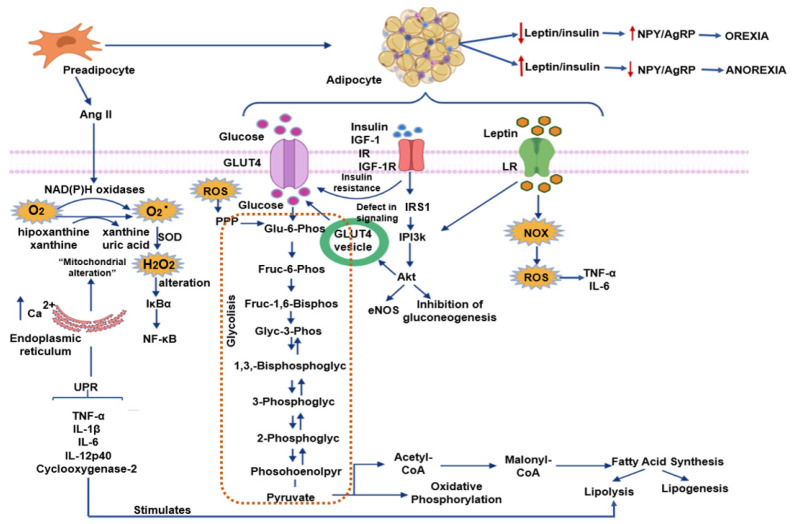
Pathways generating reactive oxygen species (ROS) and their impact on obesity Abbreviations: AgRP = agouti-related protein, Ang II = Angiotensin II, eNOS = endothelial nitric oxide synthase, H_2_O_2_ = hydrogen peroxide, IκBα = nuclear factor of kappa light polypeptide gene enhancer in B-cells inhibitor, GLUT 4 = gucose transporter type 4, IGF-1 = insulin-like growth factor 1, IGF-1R = insulin-like growth factor 1 receptor, IL = Interleucine, IR = insulin receptor, IRS 1 = insulin receptor substrate 1, LR = Leptin receptor, NF-κB = nuclear factor κ-light-chain-enhancer of activated B cells, NOX = nicotinamide adenine dinucleotide phosphate oxidase, NPY = neuropeptide Y, O^2−^ = superoxide anion, PI3k = phosphoinositide 3-kinase, PPP = pentose phosphate pathway, ROS = reactive oxygen species, SOD = superoxide dismutase, TNF-α = factor de necrosis tumoral alfa, UPR = unfolded protein response. Blue arrows indicate flow through the pathway; red arrows indicate increases or decreases.

ROS and nitrogen species (RNS) comprise hydrogen peroxide (H_2_O_2_), superoxide (O^2−^), hydroxyl radical (OH), hypochlorite (ClO^−^), nitric oxide (NO), and peroxynitrite (ONOO^−^). The most important site, where intracellular ROS are produced, is mitochondria, due to the leakage of electrons through the respiratory chain. ROS may also be produced by plasma membrane organelles and systems, such as the endoplasmic reticulum (ER), lysosomes, peroxisomes, and by cytosolic enzymes. ROS/RNS have many biological effects at low concentrations, including the defense against microorganisms that are pathogenic, which is mediated by the immune system and intracellular signaling. However, at high levels, they may damage DNA, lipids, and proteins, resulting in injury to tissues and cell death [5,6]. Some of the pathways generating ROS and their impact on obesity that are described in the following paragraphs are illustrated in Figure 1.

To maintain ROS/RNS at adequate levels, tissues have antioxidant components that work synergically to reduce free radical cytotoxicity. Naturally found antioxidant molecules comprise glutathione, ubiquinone, thioredoxin, and urate. There are also proteins, such as ferritin, transferrin, lactoferrin, and caeruloplasmin, with antioxidant properties, since they bind and hijack transition metals that may begin oxidative reactions. There are also antioxidant enzymes, which include superoxide dismutase (SOD), glutathione peroxidase (GPx), glutathione reductase, glutathione S-transferase, catalase, thioredoxin reductase, peroxiredoxins (Prx), and NAD(P)H: Ubiquinone oxidoreductase (NQO1) [5,6]. Moreover, there is a novel type of antioxidant enzymes, which, importantly participate in illnesses linked to obesity that includes the paraoxonase (PON) family [7]. Paraoxonases (aryl dialkyl phosphatases) were described as hydrolyzing enzymes of organophosphorus compounds, such as paraoxon or diazoxone insecticides. PON1 is an esterase that is generated in the liver, and it is associated with the HDL-enzyme that hydrolyzes oxidized LDL-cholesterol. It has atheroprotective capabilities [8]. The activity of PON1 is decreased in diabetes mellitus, metabolic syndrome, hypercholesterolemia, and chronic renal failure [9]. Furthermore, PON1 expression and activity can be modulated by components of the diet [10]. In addition, the rate-limiting enzyme in heme metabolism, heme oxygenase-1 (HO-1), is also an antioxidant enzyme, since it reduces oxidative stress and decreases inflammation. Recent results point to the beneficial effects of HO-1 in cardiovascular disease (CVD), and in the regulation of body weight, obesity, and diabetes [11]. Dietary antioxidants provided by the diet include vitamins C and E, and a multitude of phytochemicals. Furthermore, zinc, manganese, and selenium play an important role in regulating the activity of antioxidant enzymes [6].

### 2.1. ROS and Adipogenesis

The complex process of adipogenesis consists of a series of stages by which stem cells mature into adipocytes. ROS are involved in signal transduction and regulation of adipocyte differentiation; however, their exact function is still to be elucidated. The process is mediated by many transcription factors, cell-cycle proteins, hormones, and small molecules. Some of these pathways are regulated by receptor tyrosine kinases, AMP-activated protein kinase (AMPK), peroxisome proliferator-activated receptor γ (PPARγ), PPARγ coactivator 1α (PGC-1α), and CCAAT/enhancer-binding protein β (C/EBPβ), which are potentially sensitive to redox regulation [12].

In preadipocytes, nicotine adenine dinucleotide phosphate (NADPH) oxidase importantly produces ROS [13]. The activated insulin-like growth factor (IGF) receptor is a tyrosine kinase that controls downstream signaling pathways, including phosphatidylinositol 3-kinase (PI 3-kinase) and Ras-mitogen-activated protein kinase (MAPK) pathway, which are sensitive to ROS [14]. ROS also control MAPK activation, which is an important regulator of cell growth and differentiation through the oxidative modification of signaling proteins and the inactivation of MAPK phosphatases [15] (Figure 1). PPARγ is a fundamental regulator of adipogenesis [16]. Another adipogenesis-related redox-sensitive signaling molecule is C/EBPβ, and ROS induce a disulfide bond formation, and then dimerization of its molecule increasing its activity [17]. Moreover, oxidative stress is associated with an elevation of the expression of PPARγ, C/EBPβ [18], and PGC-1α.

**Figure 2:**
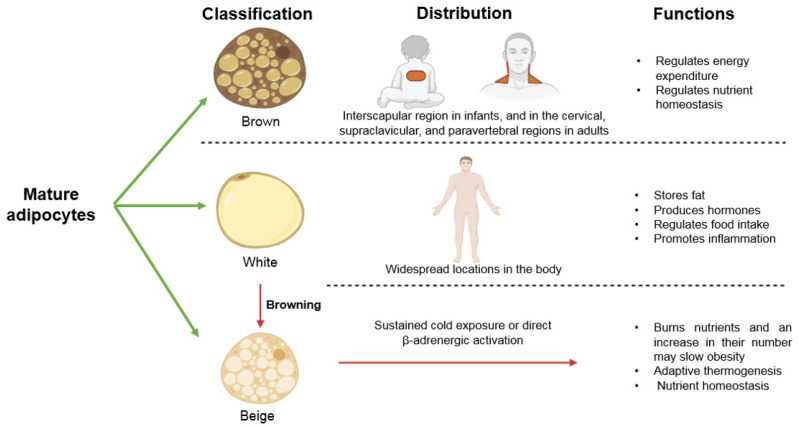
Types of adipocytes, their distribution, and their functions.

Mature adipocytes are classified into white (WAT), brown (BAT), and beige. White adipocytes have a single fat droplet and few mitochondria. They store fat, produce hormones that regulate nutrient homeostasis, participate in the regulation of food intake by secreting hormones and promote inflammation, thus playing an important role in obesity. They constitute depots with widespread locations in the body. Brown adipocytes have multiple fat droplets, many mitochondria and can be activated to oxidize fatty acids to maintain body temperature. Therefore, they regulate energy expenditure under specific conditions of physical activity and energy intake. They also regulate nutrient homeostasis and may slow obesity. They are localized in the interscapular region in infants, and in the cervical, supraclavicular, and paravertebral regions in adults. WAT is plastic, and there is an elevation in the number of adipocytes that resemble brown cells known as beige adipocytes when the individual is exposed to a cold environment or when there is the activation of β-adrenergic receptors. These beige adipocytes burn nutrients, and, an increase in their number may slow obesity. Beige adipocytes also have many fat droplets and are involved in adaptive thermogenesis and nutrient homeostasis. This plastic transformation of white to beige adipocytes is known as browning [19]. ROS could play an important role in the browning of adipose tissue, thus decreasing obesity, since natural compounds with antioxidant properties have been proven to facilitate browning [20]. In conclusion, increased ROS in fat tissue may result in altered differentiation of adipocytes and of their function and in the browning process in obesity (Figure 2).

WAT and BAT express all the components of the renin-angiotensin system (RAS), and the renin-angiotensin system is involved in obesity and insulin resistance. In the process of differentiation, preadipocytes secrete Angiotensin II (Ang II). Ang II, a vasoconstrictor agent, is a hormone that participates in many functions and is involved in several pathologies, including obesity, insulin resistance, and cardiovascular diseases [21,22]. Ang II also plays a role in adipocyte growth and differentiation and may directly stimulate leptin release from these cells. Ang II favors the synthesis of prostaglandin I2 in adipocytes, which then stimulates differentiation of adipose tissue and elevates triglyceride content [23]. Ang II is one of the most potent stimuli for the activity of NADPH oxidase (NOX), a membrane-associated multimeric enzyme, which transports electrons preferentially from cytosolic NADPH to produce a superoxide anion (O^2−^). This anion can subsequently be converted to H_2_O_2_ by superoxide dismutase [13]. Cell growth, differentiation, metabolism, host defense, and apoptosis, among other functions, are controlled by NOX proteins. NOX isoforms are present in mammalian cells: Namely, Nox1 to Nox5 and Duox1 and 2. Increased expressions of NOX2 (in macrophages) and of NOX4 (in adipocytes) are found in animal models with genetic or diet-induced obesity [24] (Figure 1).

### 2.2. Mitochondrial Activity in Obese Subjects

Aerobic organisms require oxygen to obtain the necessary energy to maintain their stability, and they possess an efficient bioenergetic system. However, oxygen can also lead to the production of free radicals and elevate ROS [25]. Cellular ROS are produced by almost all physiological processes that require oxygen, and the respiratory chain in mitochondria is considered as their main source [26]. The superoxide anion (O^−^) is the primary ROS, and it is formed by the univalent reduction of 2 oxygen molecules that are produced by the auto-oxidation of mitochondrial carriers [25]. Hydrogen peroxide (H_2_O_2_) is generated by the dismutation of superoxide anion, which is mainly catalyzed by the enzyme superoxide dismutase (SOD). The principal sites of O^−^ formation are the enzymes of the complex I [27], III [28], and II [29].

ROS production in mitochondria depends on the physiological or pathological conditions of the cell. Synthesized ROS are principally transformed and neutralized in mitochondria, and only low concentrations reach the cytosol. ROS react in these organelles with mitochondrial lipids, proteins, and DNA, and they may induce mitochondrial alterations. There is also an efficient antioxidant system in mitochondria that neutralizes ROS, allowing these organelles to remove the reactive species produced by themselves and those coming from other sources [30]. When ROS generation by mitochondria and other cellular sites increases, several constituents of the respiratory chain and Krebs cycle enzymes may lose activity, thus leading to mitochondrial dysfunction. Mitochondrial dysfunction results in several consequences in cells and the complete organism, including metabolic disorders, such as type 2 diabetes, obesity, dementia, and aging [31].

Mitochondria are central for ATP production. An excess in the availability of nutrients, causes alterations in mitochondrial number, dynamics, and morphology. It also results in abnormalities in biogenesis, ROS production, and apoptosis [32]. An elevation in glucose levels causes an increased production of ROS, which modify mitochondrial enzymes, and promote the changes in the consumption and the deposits of nutrients, thus leading to the development of metabolic disorders [31]. These changes are accompanied by alterations in the insulin signaling pathway that regulates the accumulation of free fatty acids and lipids. Mitochondrial dysfunction in adipocytes is linked to altered adipogenesis, lipolysis, fatty acid esterification, and adiponectin production [31]. Mitochondria from obese individuals decrease energy generation and diminish fatty acid oxidation [33]. They alter glucose and lipid metabolism [34] and elevate the rate of apoptosis [35]. A greater formation of lipid droplets is the result of the activation of fatty acid biosynthesis through transcriptional changes and of reprogramming to glycolysis, which is the result of mitochondrial abnormalities and decreased mitochondrial DNA [36].

The increased functions of mitochondria in obesity results in low-grade inflammation [37], and the altered responses to variations in glucose level in the hypothalamic neurons controlling energy homeostasis [38]. Damaged mitochondria may be destroyed by mitophagy [39], and they are replaced by new ones through a process that is associated with the production of ROS [40].

### 2.3. ROS and ER Stress in Obesity

The ER controls many cellular processes, such as inflammation and lipid metabolism, via the unfolded protein response (UPR) signaling pathway. It also participates in the storage of glucose, proteins, and calcium ions. High levels of Ca^2+^ are released from the ER internal deposits leading to mitochondrial dysfunction and the generation of mitochondrial ROS. Reactions by ER chaperones and oxidoreductases also generate ROS. Elevated levels of free fatty acids, including saturated fatty acids, result from the activation of the UPR in many tissues and cell types. Moreover, changes in the lipid composition of the ER activate the UPR, and these results in modifications of the activity of the sarco-/endoplasmic reticulum calcium ATPase and the subsequent disequilibrium in homeostasis [41]. Increases in the activity of pro-inflammatory genes, such as those encoding tumor necrosis factor alpha (TNF-α), interleukins IL-1β, IL-6, IL-12p40, and cyclooxygenase-2, from the activation NF-κB (nuclear factor κ-light-chain-enhancer of activated B cells)—an important transcription factor of macrophages, which occurs by an increase in the activity of the UPR [42,43].

### 2.4. ROS and Lipolysis and Lipogenesis in Obesity

The enzymes in charge of lipolysis and lipogenesis are ROS-sensitive [44,45]. Lipogenesis is the reaction by which fatty acids that form lipoproteins are esterified with glycerol to constitute triglycerides. It is catalyzed by the enzyme lipoprotein lipase (LPL) [46,47,48]. The products of this reaction are then stored in lipid droplets. Lipolysis, which is mediated by the hormone sensitive lipase (HSL), is the breakdown of stored TG [46,47,48]. The signaling cascade of norepinephrine (NE) and other hormones that include cyclic adenosine monophosphate (which activates protein kinase A (PKA), decreases the expression and/or activity of HSL [49,50]). NE also controls the phosphorylation of perilipin A which helps in the translocation of HSL from the cytosol to the lipid droplets [51]. Alterations in LPL expression and/or activity also induce obesity and hypertriglyceridemia [52]. NEFAs and glycerol are generated when triglycerides are hydrolyzed by LPL from circulating chylomicrons and VLDL. These molecules are then re-esterified and deposited in adipocytes [53]. Lipolisis is increased by IL-1, IL-6 y TNF-α, and it then stimulates the de novo synthesis and the secretion of hepatic fatty acids [54].

### 2.5. ROS and Inflammation in Obesity

Phagocytic leukocytes, including macrophages, monocytes, neutrophils and eosinophils, which invade tissues produce oxidizing agents. The increased size and number of adipocytes trigger the invasion of fat tissue by these cells. ROS induce the mechanism of inflammation by activating NF-κB and elevating the transcription of cytokine-producing genes. The inflammatory response is increased by the liberation of cytokines [55]. A state of systemic inflammation is induced by the growth of the mass of adipose tissue as a response to an increase in secretory factors that come from the preadipocytes and macrophages. This inflammation has as a consequence endothelial dysfunction, which is present in CVD when metabolic dysfunction is present [56].

Anti- and pro-inflammatory cytokines, hormones, growth factors, complement factors, and matrix proteins are secreted by adipocytes and are known as adipocytokines [57]. They include anti-inflammatory molecules, such as adiponectin, transforming growth factor beta (TGFβ), IL-10, IL-4, IL-13, and IL-1, receptor antagonist (IL-1Ra), and apelin. They also include pro-inflammatory molecules, such as tumor necrosis factor-α (TNF-α), IL-6, leptin, visfatin, resistin, Ang II, and plasminogen activator inhibitor1 [58]. When obesity is present, there is an increase in the number and size of the adipocytes, and the secretion of various pro-inflammatory molecules is promoted, thus propitiating the pathological state [59].

The activity of inflammasomes is also controlled by the NF-κB signaling pathway, which induces the transcriptional expression of NOD-, LRR- and pyrin domain-containing protein 3 (NLRP3) [60]. Excess nutrition induces NF-κB signaling pathways in adipocytes and in skeletal muscle cells by interfering with mitochondrial function leading to the overproduction of ROS [61]. Hydrogen peroxide (H_2_O_2_) affects the degradation of the nuclear factor—enhancing kappa light chains of activated B cells (IκBα), an NF-κB inhibitor, through tyrosine phosphorylation [62]. Many enzymes, such as NADPH oxidase, cyclooxygenase-2, and arachidonate 5- and 12-lipoxygenases, are activated after DNA binding. This enhances overproduction of ROS or the liberation of nitric oxide synthases that leads to the production of NRS. This potentiates ROS-induced damage [63].

Free fatty acids activate the Toll-like receptor (TLR) pathway, a family of surface receptors that are present in all cells. It regulates the expression of numerous inflammatory factors. The TLR2 and TLR4 pathways, activated by FFAs, play an important role in insulin resistance and vascular dysfunction [64,65]. The presence of ROS up-regulates the expression of TLR2 and 4 [66]. Some proposed mechanisms by which free fatty acids carry out their function by binding to TLRs are the formation of ceramides, the stimulation of the activity of several serine/threonine kinases, among which the protein kinase C (PKC), the increase in the production of oxygen free radicals, due to the stimulation of the activity of NOX and the activation of the factor NF-kappaB may be cited. These pathways probably work synergistically [64].

### 2.6. Oxygen Reactive Species and Adipokines Regulating Appetite

ROS play an important role as signaling molecules in the central nervous system. They participate in the regulation of metabolism and food intake by acting on the hypothalamus. They regulate proopiomelanocortin (POMC) neurons and agouti-related protein/neuropeptide Y neurons (NPY/AgRP) [67,68]. The principal substrate of metabolism in POMC neurons is glucose, and when these cells are activated, food intake is reduced, and energy expenditure is increased. The main substrates for NPY/AgRP neurons are fatty acids, and when they are activated, food intake is increased, and energy expenditure decreased [69].

The hypothalamus is rich in mitochondrial ROS. The metabolism of glucose, lipid, insulin, and leptin in POMC and NPY/AgRP neurons generates ROS during a positive energy state, via Ca^2+^ influx and mitochondrial activity. The NPY/AgRP neurons are activated in a negative energy state, this results in a decrease in the level of ROS. Therefore, oxygen production must be balanced to maintain homeostasis [70].

#### 2.6.1. Leptin

Leptin was the first adipokine to be described, and it plays an important role in controlling the equilibrium between the expenditure of energy and the ingestion of food. It is a pleiotropic molecule influencing several organs and systems. Fat cells produce leptin, which regulates hunger and appetite, and therefore, food intake. Even if there are high leptin levels, the response to leptin, which is normally linked to satiety, is disrupted, and individuals continue to consume calorie-rich food [71].

Obesity is linked with an elevation of the levels of leptin and chronic immune-mediated inflammation. Leptin also has effects on the immune system, since it increases macrophage proliferation, and it activates the NADPH oxidase via the PI3K/PKC pathway. Thus, leptin may contribute to atherosclerosis development in obese patients [72]. It also stimulates the secretion of pro-inflammatory cytokines, such as TNF-α and IL-6, in endothelial cells [73]. Leptin also favors the Th1-type of activity, which reduces the levels of tryptophan. Serotonin and melatonin, which are derivatives of tryptophan, induce satiety through diverse mechanisms. Therefore, the suppression of leptin release and of the Th1-type activity by antioxidants elevates serotonin and melatonin concentrations [74]. In the hypothalamus, leptin elevates the norepinephrine and epinephrine levels contributing to hypertension [75].

#### 2.6.2. Adiponectin

Adiponectin is constituted by a globular domain that is structurally resembles TNFα and a collagen domain. In obese patients and in subjects with type-II diabetes mellitus, serum levels of this hormone are diminished. Adiponectin reduces serum triglycerides, stimulates fatty acid oxidation, elevates insulin sensitivity. The anti-inflammatory effects of adiponectin are due to the reduction of the level of NF-κB, which controls more than 150 target genes. Among the genes that it controls, those of pro-inflammatory enzymes, such as cyclo-oxygenase-2, cytokines, chemokines, immunoreceptors, and adhesion molecules, are included [75]. When the concentrations of adiponectin are increased, the production and activity of TNF-α, IL-6, and interleukine-8 (IL-8) are reduced, and the expression of cellular α adhesion molecule 1 (ICAM-1), vascular cellular adhesion molecule 1 (VCAM-1), and E-selectin are decreased. There is also an elevated synthesis of interleukin 10 (IL-10), an anti-inflammatory cytokine [76]. Interleukines IL-1, IL-6 y TNF-α nuclear factor—enhancing kappa light chains of activated B cells increase lipolisis and stimulating the de novo synthesis and the secretion of hepatic fatty acids [54,77,78]. Therefore, there is an association between the increase in the levels of serum triglycerides and cholesterol [54]. The benefic effects of adiponectin are observed when its concentration is increased by including fish oil or soy in the diet, or by using tiazolininediones or peoxisomal proliferation receptor gamma PPARγ [79,80,81,82].

Increased OS and decreased adiponectin participate in pathological conditions, such as the insulin resistance related to obesity, and they elevate the risk of CVD. Different nuclear receptors participate in this pathogenesis. The transcription of the target genes in response to metabolic and nutritional substrates is controlled by nuclear hormone receptors (NRs), such as PPARγ, which promote the transcription of adiponectin and antioxidant enzymes. In contrast, the mineralocorticoid receptor mediates the effects of aldosterone and glucocorticoids and promotes the generation of OS in adipocytes [83].

### 2.7. Oxidative Stress, Iron Metabolism and Hepdicin in Obesity

Obesity is characterized by chronic low-grade inflammation that leads to the production of inflammatory cytokines, increasing OS and elevating hepcidin secretion in hepatocytes and macrophages [84]. Hepcidin is a peptidic hormone, mainly produced by the liver, which regulates iron metabolism [85,86,87]. The increased OS contributes to the development and progression of various diseases that influence obesity.

There is an association between obesity and iron deficiency in which hepcidin participates [88,89]. Serum hepcidin is significantly elevated in obese people [90]. Iron induces OS, since it is a metal with redox activity. It also enhances ER stress, inflammation, and endocrine dysfunction of adipose tissue. Mechanisms of toxicity mediated by iron modify aspects of the pathogenesis of obesity, resulting in its exacerbation. Free intracellular iron is cytotoxic, promoting the Fenton reaction, and exacerbating OS [91]. Ferritin is of crucial importance in the protection of the liver against oxidative damage. When there is insufficient positive regulation of ferritin, free iron can exert its pro-oxidant and cytotoxic effects. These molecular events lead to positive feedback in ROS production and contribute to the pathophysiological alterations in obesity. Hepdicin also targets the intestinal absorption of iron and the function of ferroportin within the cell, thus playing a central role in iron homeostasis [87,92,93].

Overproduction of hepcidin is a possible cause of obesity-related hypoferremia [94,95]. Excessive secretion of this protein leads to iron sequestration in cells of the reticuloendothelial system. The latter results in increased iron content in adipose tissue, which programs adverse effects and an overload of local iron.

### 2.8. Sympathetic Nervous System and Obesity

The activity of the sympathetic nervous system (SNS) is linked to energy balance even if the contribution of the SNS to energy expenditure is small, accounting for only 5% [96]. Since the brain has a high oxygen consumption and is rich in lipids, it is very vulnerable to OS, and damage induced by OS has a strong potential to impact normal CNS functions [97]. Over-activation of the SNS is associated with the excess production of ROS and is linked to metabolic disorders, including obesity, diabetes mellitus type 2, hypertension, and CVD [71,98]. OS and inflammation in the hypothalamic paraventricular nucleus (PVN), mediate sympathetic excitation, particularly in cardiovascular diseases [99]. Medications with antagonistic effects to those of the SNS increase food intake, decrease resting metabolic rate and thermogenic responses, while sympathomimetic medications have the contrary effects. Fasting blocks SNS activity, while ingesting food increases its activity. Therefore, SNS predominance and diminished SNS responsiveness in the basal state favors obesity [100]. Weight loss inhibits SNS overactivity in obese subjects. Activity in the SNS is linked to visceral fat rather than with total fat mass. Lipolysis in visceral fat is mediated by the action of catecholamine on the beta(3)-adrenoceptors. Moreover, alterations in the hypothalamo-pituitary-adrenal axis have been related to the distribution of central fat [96]. In addition, a pro-inflammatory state is promoted by the activity of the SNS by the synthesis and liberation of IL-6, which promotes an acute phase response [101].

## 3. Natural Products with Anti Obesogenic Effects

Natural antioxidants in foods have recently gained attention because of their capacity to counteract the deleterious effects of an excess of free radicals and pathologies associated with them. Insufficient burning of calories and alterations in the regulation of food intake behavior may be caused by insufficient exposure to oxidizing compounds. Diets rich in fruits and vegetables reduce obesity, metabolic syndrome, type 2 diabetes, CVD, inflammation, neurological disorders, and cancer [102]. The mechanisms of action include antioxidant and/or anti-inflammatory properties, such as the kidnapping of free radicals and changes in gene transcription through the induction or inhibition of transcription factors.

Another cytoprotective mechanism, used by natural compounds against excess ROS, is the control of the signaling pathway of the nuclear transcription factor erythroid 2p45 (NF-E2)-related factor 2 (Nrf2) [103]. The anti-inflammatory activity of some chemopreventive/cytoprotective agents correlates with the ability to induce antioxidant gene expression. Inactive Nrf2 is present in the cytoplasm linked to the Keap 1 protein. When the Nrf2-Keap1 dissociates, due to changes in the cellular redox state, Nrf2 translocates to the nucleus and interacts with Maf proteins constituting heterodimers that interact with antioxidant responsive elements (ARE) or electrophile responsive elements (EpRE). These elements are found in the promoter/enhancer regions of genes that encode for most of the phase II detoxification antioxidant and detoxifying enzymes [103]. Detoxifying enzymes include heme oxygenase-1 (HO-1), glutathione peroxidase (GPx), glutamate cysteine ligase catalytic (GCLC) and modifier (GCLM) subunits, glutathione S-transferase (GST), NAD(P) H:quinone oxidoreductase 1 (NQO1), and superoxide dismutase (SOD) [104].

Overexposure to food antioxidants can also lead to adverse effects. Antioxidant stress in young ages participates in the development of adiposity later in life. Some food preservatives, considered as food antioxidants, including sodium sulfite, sodium benzoate, some spice compounds, and natural colorants (such as curcumin), diminish the liberation of leptin in murine 3T3-L1 adipocytes—in which obesity-like inflammation was induced which co-incubation with lipopolysaccharide (LPS). Therefore, consumption of antioxidant supplemented food could result in diminished leptin liberation, giving rise to an obesogenic environment [105].

### 3.1. Groups of Compounds with Antioxidant Properties Found in Natural Plants

**Figure 3:**
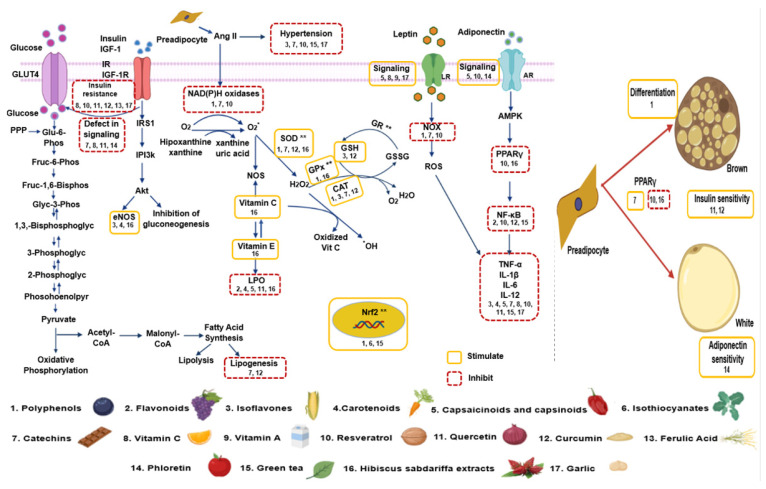
Summary of the interaction of antioxidant groups found in natural plants and the different metabolic pathways to induce anti obesogenic effects. The numbers correspond to the natural product and the “**” correspond to the enzymes stimulated by Nrf2. Abbreviations: Ang II = Angiotensin II, eNOS = endothelial nitric oxide synthase, H_2_O_2_ = hydrogen peroxide, GLUT 4 = glucose transporter type 4, GR = glutathione reductase, GSH = glutathione, GSSG = oxidized glutathione, IGF-1 = insulin-like growth factor 1, IGF-1R = insulin-like growth factor 1 receptor, IL = Interleukin, IR = insulin receptor, IRS 1 = insulin receptor substrate 1, LR = Leptin receptor, NF-κB = nuclear factor κ-light-chain-enhancer of activated B cells, NOS = nitric oxide synthase, NOX = nicotinamide adenine dinucleotide phosphate oxidase, O^2−^ = superoxide anion, PI3k = phosphoinositide 3-kinase, PPAR = peroxisome proliferator–activated receptor, PPP = pentose phosphate patway, ROS = reactive oxygen species, SOD = superoxide dismutase, TNF-α = factor de necrosis tumoral alfa.

Important groups of compounds found in plants with antioxidant properties include (a) polyphenols such as phenolic acids, stilbenes, flavonoids (flavonols, flavanols, anthocyanins, flavanones, flavones, flavanonols, and isoflavones), chalcones, lignans, and curcuminoids (b) carotenoids, (c) capsaicinoids and casinoids, (d) isothiocyanates, and (e) catechins and will be described in the following sections. A summary of the metabolic pathways upon, which these different antioxidant groups of compounds act, is shown in Figure 3.

#### 3.1.1. Polyphenols

Polyphenols constitute the most abundant phytochemicals of plant origin. Polyphenols play a protective role against pathologies in which OS intervenes, such as metabolic disorders that include obesity, CVD, and cancer [106,107]. However, their mechanisms of action are still unclear. They can be found in fruits, vegetables, whole cereals, coffee, cacao, and tea. They have the potential for inducing weight loss and have been included in dietary strategies to abolish OS to prevent obesity, by acting on mitochondrial dysfunction, inflammation, and over-activation of the sympathetic nervous system [3,108]. Even when the mechanisms of action of polyphenols are known, each polyphenol produces different physiological effects that correlate with its chemical structure, bioavailability, and metabolism. Although polyphenol intake is of approximately 1000 mg/day in European populations [109], these molecules are poorly absorbed, and their metabolism is fast.

Polyphenols kidnap free radicals, increase the activity and expression of antioxidant enzymes and inhibit those of ROS-producing enzymes. They also chelate metals. The most abundant polyphenols are flavonoids comprising more than 6000 types that have been identified and characterized by their structure. The antioxidant activity of polyphenols resides in their scavenger ability and their capacity to inhibit ROS-generating enzymes, such as NOX and iNOS. Polyphenols neutralize ROS by donating an electron or hydrogen atom, they have properties as chelators, they exert co-antioxidant activity with essential vitamins, they inhibit the oxidase and arachidonic acid pathways, and they up-regulate SOD, CAT, and GPX enzymes [110,111]. They also promote the expression of antioxidant enzymes that include those involved in glutathione synthesis and phase II drug metabolism, through the regulation of the Nrf2/Keap1 pathway.

These molecules also possess anti-inflammatory, anti-diabetic and anti-cancer properties. They modulate inflammation caused by elevations in the number and size of adipocytes. Polyphenols induce the synthesis of pro-inflammatory molecules, such as cytokines, they block TLR and regulate several inflammatory-related pathways, such as the NF-κB, MAPK, PI3K/AkT, IKK/JNK, and JAK/STAT, thus regulating the immune response. They also interfere with immune cell regulation, pro-inflammatory cytokine synthesis, and gene expression. Polyphenols are also nutraceuticals that prevent hypothalamic inflammation and regulate the energy balance [112].

Polyphenols also control adipocyte differentiation and lipid metabolism, since they decrease the activity of the pancreatic lipase and the permeability of the intestine, and through their interaction with the gut microbiota [108,113,114]. They also inhibit lipid accumulation in 3T3-L1 cells and modify the activity of pancreatic lipase [4]. Furthermore, some polyphenols can activate sirtuin 1 (Sirt1) and are potential inducers of mitochondrial biogenesis via deacetylation-mediated PGC-1α activation [114].

Polyphenols stimulate mitochondrial biogenesis and diminish mitochondrial dysfunction [115]. Resveratrol inhibits cAMP phosphodiesterases, elevates cAMP, and stimulates the cAMP/CaMK/AMPA pathway to deacetylate and activate PGC-1α via NAD^+^/Sirt1. It also activates protein kinase C epsilon (PKCε) and AMPK. It also increases NAD^+^ levels, and stimulates mitochondrial function, biogenesis, and dynamics [116]. Flavones, isoflavones, curcumin, and hydroxytyrosol (3,4-dihydroxyphenylethanol), enhance mitochondrial biogenesis by increasing the expression of Sirt1/AMPA/PGC-1α, complex IV in the electron transport chain (ETC), and the mitochondrial transcription factor (TFAM) [117].

Unfortunately, there are still no prospective studies in humans, and the link between polyphenols, obesity, and chronic diseases still needs to be clarified [118]. There are only a few clinical trials that address the short-term effects of a single molecule or of food extract supplementation on obesity and obesity-related diseases. In these studies, the markers of OS and inflammation, glucose tolerance, and CVD risk factors were evaluated, and a positive role of these molecules was found [110,111].

Polyphenols are classified into different categories that relate to their chemical structure and include phenolic acids, stilbenes, flavonoids (flavonols, flavanols, anthocyanins, flavanones, flavones, flavanonols, and isoflavones), chalcones, lignans, and curcuminoids.

#### 3.1.2. Flavonoids

Flavonoids are phenolic phytochemicals that are important constituents of the human diet [116,119,120]. They are divided into subgroups based on the degree of oxidation of the rings that confirm their structure. There exist six main subgroups: (1) Flavonols, like kaempferol and quercetin, (2) flavanols, like epicatechin EC, ECG or EGCG; (3) flavones, like apigenin; (4) isoflavones, like genistein; (5) flavanones, like hesperetin, raringenin); and (6) anthocyanins, like cyanidin or delphidin, malvidin [121].

Flavonoids may have therapeutic potential for diseases, such as cancer, ischemic heart disease, and atherosclerosis. They improve health through biological functions, such as the scavenging of ROS, inducing apoptosis, and inducing antitumorigenic activity. In their structure, they have phenolic hydroxyl groups, which makes them strong antioxidants acting against ROS that participate in the initiation of lipid peroxidation [119,120]. Furthermore, flavonoids are soluble inhibitors of the breakage of chains of molecules by peroxidation, and they scavenge intermediate peroxyl and alkoxyl radicals. Several flavonoids are also able to block the expression of NF-κB–dependent genes [122].

#### 3.1.3. Isoflavones

Isoflavones are found in legumes, grains, and vegetables; however, soybeans are the main source of these polyphenols in human diet. Isoflavones include genistein, daidzein, and glycitein. Evidence also suggests that they may protect against obesity and its co-morbities. They have anti-adipogenic and anti-lipogenic effects, which are due to their interaction with estrogen receptors and PPARγ, which allows them to control insulin sensitivity, fatty acid metabolism, and adipose development. They may also have other protective mechanisms, such as decreased OS and inflammation [123].

Genistein may block the damaging effects of ROS, since it is able to act as an antioxidant that chelates metals and elevates reduced/oxidized glutathione ratio (GSH/GSSG) and mitochondrial membrane potential. Genistein also restores antioxidant enzyme activities and reduces the generation of ROS, pro-inflammatory cytokines, and iNOS and endothelial NOS (eNOS). It reduces inflammation, by activating JNK and inhibiting NF-κB, TNF-α, and IL-6 secretion [124].

Isoflavone supplementation to healthy women reduced DNA oxidative damage and increased total plasma antioxidant capacity [125]. In overweight, diabetic subjects, isoflavone intake increased plasma total antioxidant capacity and GSH levels, and reduced oxLDL and isoprostane 8-iso-PGF2α, [126]. Isoflavones exert estrogen-like effects, and therefore, have been classified as phytoestrogens being useful in the treatment of hormone-dependent cancers [127,128]. Genistein supplementation in postmenopausal women diminishes body mass index, body fat mass, waist size, and increases blood HDL [129,130].

#### 3.1.4. Carotenoids

Carotenoids are found in plants, microorganisms, and few animals and are important antioxidant compounds. They have pro-vitamin A activity, especially β-carotene, α-carotene, and β-cryptoxanthin. Carotenoids protect against metabolic syndrome, CVD and cancer, through their role as an antioxidant and anti-inflammatory agents [131,132]. Obese subjects have low serum carotenoid levels [133], and consumption of vegetables and fruits that contain large amounts of carotenes, including carrots, pumpkin, tomato, broccoli, spinach, apricots, and mandarins, is inversely correlated to inflammation, OS, endothelial dysfunction, cardiovascular mortality and overall mortality in the elderly [134].

The carotenoid produced by the microscopic algae Astaxanthin, which is present in salmon, crabs, and lobster, blocks lipid peroxidation and enhances antioxidant defense activity, in overweight and obese adults [135]. Supplementation of highly concentrated β-cryptoxanthin elevates adipokine levels in moderately obese postmenopausal women [136]. Lycopene or tomato intake diminishes OS in diabetic patients, but it did not reduce the risk for developing diabetes [137]. Lycopene supplementation reduced systolic blood pressure in mildly hypertensive subjects [138]. Supplementation studies using other carotenoids demonstrated a positive action of these compounds on redox balance, inflammation, and hypertension. The combination of α-carotene, β-carotene, lutein/zeaxanthin, and cryptoxanthin is negatively associated with the incidence of hypertension [139].

However, most studies using supplementation did not show a beneficial effect of β-carotene, which is the most used carotenoid in supplementation on CVD, metabolic syndrome [5], and cancer risk [140], and it is reported that it increased the risk of lung cancer and fatal coronary heart disease [141,142].

#### 3.1.5. Capsaicinoids and Capsinoids

Capsaicinoids and casinoids are alkaloids found in red hot peppers and sweet peppers. They play physiological and pharmacological roles, including antioxidant, anti-inflammatory, anti-obesity, and anti-cancer effects [143]. The ingestion of capsaicinoids elevates the expenditure of energy and the oxidation of lipids, reducing appetite and energy intake and helping in weight loss [144]. The molecular mechanisms of action are still not well known, although stimulation of the transient receptor potential vanilloid type-1 (TRPV1) may be the cause of many of the benefic effects observed [144]. Capsaicin also reduces the inflammation that is induced by obesity by decreasing TNF-α, IL-6, IL-8, and MCP-1 levels [145], and elevating adiponectin levels, which are important for the insulin response [146].

#### 3.1.6. Isothiocyanates and Catechins

Isothiocyanates (ITCs) are compounds with chemopreventive actions that are present in cruciferous vegetables, including broccoli, watercress, Brussels sprouts, cabbage, and cauliflower. ITC contain sulforaphane and phenethyl, which may explain their properties. Although ITCs are not antioxidants themselves, they show strong antioxidant effects by transcriptionally activating Nrf2 [147].

Cathechins are the main flavonols present in tea, being also present in cocoa, grapes, and red wine [109]. Catechins reduce OS and inflammation, by elevating the expression of SOD and catalase and by reducing the activities of Nox, iNOS, TNF-α, and NF-κB [148]. Supplementation with epigallocatechin gallate, one of the most common catechin, increases thermogenesis, improves glucose tolerance, and elevates the expression of PPARγ, in rats fed with a high-fat diet [110]. In streptozotocin-diabetic rats, it induces a hypoglycemic condition accompanied by a healthier lipidic profile [138]. Enriched diets with Epicatechin decreased IGF-1 levels and increased the lifespan of diabetic mice and of Drosophila melanogaster [149]. In humans, catechins diminish obesity, blood pressure, LDL-cholesterol, and CDVD risk factors. Catechin-rich beverages, such as green tea, reduce obesity and decrease glucose levels in patients who have type 2 diabetes [150].

#### 3.1.7. Vitamins, Oxidative Stress, and Obesity

Vitamins are found in many fruits and vegetables. A deficiency in vitamin C is a common characteristic of obese individuals [151]. There is a negative correlation between vitamin C levels and body mass index, waist-to-height ratio, and leptin concentrations [152]. Fifteen enzymes have as a cofactor vitamin C, and this vitamin has antioxidant activity being a donor of electrons. It is an important scavenger of free radicals and it protects tissues against OS diminishing inflammation [153]. Vitamin C inhibits mature adipocyte formation and cell growth, it blocks lipolysis, controls glucocorticoid liberation from adrenal glands, inhibits glucose metabolism and leptin secretion—resulting in reduced hyperglycemia and a decrease glycosylation [154,155].

Although vitamin E status was not associated with any markers of obesity [152], obese people with metabolic syndrome need more vitamin E than normal people because their weight and other problems cause increased OS. However, those same problems cause a reduction in the employment of vitamin E [156].

Vitamin A levels are directly associated with leptin [152], and higher vitamin A levels are present in women without obesity. There is an association between vitamin D deficiency and overweight [157]. Despite these reports, available data do not support vitamin supplementation in obesity [3].

### 3.2. Natural Products with Antioxidant Effects

#### 3.2.1. Resveratrol

Resveratrol (3,4′,5-trihydroxystilbene), is a small phytoalexin that is a polyphenolic compound, present in the skin and seeds of red grapes, red wine, peanuts, apples, and groundnuts [158,159]. Some spermatophytes, like grapevines, produce it when they are injured or attacked by fungus. Resveratrol avoids the appearance of several diseases, such as obesity, metabolic disorders, type 2 diabetes, CVD, cancer, and aging, through its antioxidant and anti-inflammatory actions [160]. It also has vasoprotective effects in animal models [161]. Although its mechanisms of action are not still completely understood [162,163], they include changes in mitochondrial activity, blockage of lipid accumulation, reduction of inflammation, improvement of insulin signaling and modulation of redox balance. Resveratrol attenuates diet-induced OS in epididymal WAT by decreasing the levels of Sirt1 and manganese superoxide dismutase (SOD2) [164]. Resveratrol chelates free copper ions and remove copper ions, which are bound to ApoB, the main apolipoprotein of chylomicrons, VLDL, IDL, and LDL particles [9].

In obese subjects, supplementation with resveratrol triphosphate decreased biochemical parameters of OS and modulated the expression of redox-sensitive genes in blood cells [165]. Resveratrol diminished the expression of inflammatory mediators (TNF-α, IL-6, COX-2) and blocked NF-κB signaling. In preadipocytes, resveratrol reduced PPARγ expression and elevated the expression of genes that control the activity of mitochondria, such as SIRT3, uncoupling protein 1, and mitofusin 2 [166]. It also promoted lipolysis and apoptosis, decreasing lipogenesis and proliferation of mature human adipocytes [106,167].

In healthy obese men, resveratrol lowers OS, and it has effects that resemble those of calorie restriction [166]. Resveratrol supplementation improves insulin sensitivity in type 2 diabetes. The effective resveratrol concentration for improvement in diabetes is still in doubt, since the doses used in studies is variable [168,169].

Resveratrol also provides cardiovascular benefits, by elevating serum adiponectin, preventing plasminogen activator inhibitor-1 (PAI-1), and blocking the atherothrombotic signals in blood mononuclear cells [170]. In type 2 diabetes and hypertensive patients with coronary artery disease, it regulates the mRNAs related to inflammation and cytokine expression, in peripheral blood mononuclear cells [171]. Resveratrol suppressed the Ang II/AT1R axis and enhanced the protective axis of the RAS system [172]. Resveratrol also decreases Nox activity and induces NQO1 and glutathione S-transferase-1P expression in mononuclear cells [173]. Resveratrol also increases PON1 gene expression and activity in different cell types [174]. It prevents the disruption of the intestinal barrier, which is mediated by OS [175].

Our group has studied the effects of resveratrol potentiated by small doses of quercetin (RSV + QRC) in obese rats from a rat model of metabolic syndrome induced by high sucrose ingestion. The RSV + QRC mixture prevented the elevation in systolic blood pressure, insulin levels, insulin resistance index homeostasis model (HOMA), triglycerides, leptin, and adiponectin, in these rats. The sucrose treatment increased carbonylation and lipid peroxidation, while glutathione (GSH) and the total antioxidant capacity were diminished, and RVS + QRC restored their levels. In the metabolic syndrome rat model, catalase, superoxide dismutase isoforms, peroxidases, glutathione-S-transferase, glutathione reductase, and the expression of Nrf2 were reduced, and RVS + QRC reversed these effects. RSV + QRC also reduced OS in fatty liver in the MS rats by improving the antioxidant capacity and by the over-expressing Nrf2, which elevates the antioxidant enzymes and recycled GSH [176]. RSV + QRC administration also reduced body mass, central adiposity, insulin, triglycerides, non-HDL-C, leptin, adiponectin, monounsaturated fatty acids (MUFAs), and non-esterified fatty acids (NEFAs) in metabolic syndrome rats that were obese and up-regulated SIRT 1 and SIRT 2 expression in abdominal WAT [177]. We also studied the control of the expression of UCP1, -2, and -3 in abdominal WAT from metabolic syndrome rats treated with (RSV + QRC). Uncoupling proteins (UCPs) are mitochondrial anion carriers that participate in controlling body temperature and energy balance regulation. We found that in metabolic syndrome rats, the mostly-expressed isoform was UCP2, low levels of UCP3 were present, and UCP1 was undetectable. RSV + QRC increased UCP2 mRNA in control and metabolic syndrome rats, and the elevation was associated with an increase in oleic and linoleic fatty acids. Metabolic syndrome rats had an enhanced expression of peroxisome proliferator-activated receptor, and its protein levels were increased RSV + QRC [178].

#### 3.2.2. Quercetin

Quercetin (3,5,7,3′,4′-pentahydroxy flavone) is a flavonol that is widely distributed in fruits and vegetables. It is abundant in apples, onions, scallions, broccoli, and teas. It is a major component in Gingo biloba extract and has multiple biological functions, including antioxidative, anti-inflammatory, and anti-mutagenic activities. Quercetin decreases inflammation by controlling the release of TNF-α and the levels of nitric oxide and IL-6 [179]. It decreases OS, by blocking the expression of metalloproteinase 1 and the oxidation of LDL, thus inhibiting the disruption of atherosclerotic plaques and stabilizing the plaque.

Quercetin decreases inflammation and reduces insulin resistance, by elevating the expression of the GLUT4 glucose transporters. It diminishes JNK phosphorylation, and the expression of TNF-α and iNOS in skeletal muscle. It reduces adipogenesis in L6 myotubes [180]. In primary human adipocytes, it stimulates insulin sensitivity and reduces inflammation, by decreasing the expression of IL-6, IL-1β, IL-8, and MCP-1 [181]. In mice and rats fed a diet rich in calories, quercetin lowers circulating glucose, cholesterol, insulin, and triglyceride levels, and elevates the secretion and expression of adiponectin [110]. Quercetin supplementation reduces the inflammatory state in the adipose tissue of obese Zucker rats, and it diminishes dyslipidemia, hypertension, and hyperinsulinemia. In overweight-obese subjects, quercetin supplementation reduces plasma TNF-α and oxLDL [182].

Although quercetin suppressed OS in obese rodent models [183,184,185], it had no effect on OS and antioxidant capacity in obese subjects [186]. Future research is needed to elucidate the bioavailability and bioactive effects of quercetin to reduce obesity.

#### 3.2.3. Curcumin

Curcumin is a polyphenol extracted from the rhizome of the plant Curcuma longa. It has anti-obesity, anti-diabetic, anti-inflammatory, and anti-cancer properties [187]. It elevates glutathion. The underlying mechanisms of action are through the down-regulation of redox-sensitive transcription factors NF-kB by decreasing ERK1/2 and p38 MAPK [188,189], inflammatory cytokines, and growth factors.

At a cellular level, curcumin promotes mild oxidative and metabolic stress, which results in an adaptive response, in which lipid metabolism enzymes and antioxidant enzymes, including catalase, MnSOD, and HO-1, are involved [190]. In adipose tissue, curcumin blocks the infiltration by macrophages and the activation of NF-κB [191]. In the liver, it enhances high fat diet-induced insulin sensitivity and diminishes obesity, blocking lipogenesis [192]. Curcumin also has an anti-cancer activity that, may be due to its estrogen-like effects [193].

#### 3.2.4. Ferulic Acid and Phloretin

Ferulic acid is a phenolic acid that can be found in apples, oranges, chocolate, whole wheat, sage, and oregano, which prevents hyperlipidemia induced by high fat and OS. It controls insulin secretion and regulates the activities of antioxidant and lipogenic enzymes [112].

Phloretin, a natural antioxidant product from apple tree leaves and Manchurian apricot, reduces obesity and improves metabolic homeostasis. The activities of phloretin to prevent and treat obesity have been studied in a high-fat diet-induced obesity mouse model. Although phloretin does not cause weight loss in obese animals, it blocks weight gain. Phloretin improves glucose homeostasis and insulin sensitivity and decreases hepatic lipid accumulation. It favors the expression of the gene of adiponectin in WAT. In addition, phloretin treatment elevates the expression of fatty acid oxidation genes [194].

### 3.3. Plants with Antioxidant Effects

#### 3.3.1. Green Tea

Green tea, which is prepared from leaves of *Camellia sinensis*, is one of the most popular beverages. Green tea is rich in many catechin polyphenols, such as (-)-epicatechin, (-)-epicatechin-3-gallate, (-)-epigallocatechin, and (-)-epigallocatechin-3-gallate (EGCG). The main polyphenol in green tea is EGCG, which has antioxidant, anti-inflammatory, antiproliferative, and antithrombogenic effects. It also has benefic effects on endothelial function [195]. It controls redox-sensitive transcription factors, such as NF-κB, Nrf2, and AP-1 [196]. Green tea extracts decreased blood pressure, inflammatory biomarkers, and OS, and elevated insulin sensitivity in obese, hypertensive patients [197]. Drinking green tea or its extract supplementation, causes a decrease in body weight and lipid peroxidation in obese subjects with metabolic syndrome [198].

#### 3.3.2. Hibiscus Sabdariffa Extracts

The flowers of Hibiscus Sabdariffa (HSE) contain many chemicals, including: Polyphenols, flavonoids (including anthocyanins, delphinidin, hibiscetin, quercetin, and gossypetin), protocatechuic acid alkaloids, L-ascorbic acid, among others. Of these, anthocyanin flavonoids and protocatechuic acid are antioxidant and anti-diabetic compounds [199,200]. There is considerable variation from one species to another in the anthocyanins found in the plant, and their efficacy differs significantly. This variation is due to the level of methylation of the hydroxyl groups, to differences in the number of hydroxyl groups, the characteristics and number of the sugars s that are bound to the anthocyanidin molecule and the position of their attachment, and the variety and number of aliphatic or aromatic acids bound to the sugars [199,200].

The HSE extract also controls adipogenesis by inhibiting the expression of the adipogenic transcription factors C/EBPα and PPARγ, through the PI3-K and MAPK pathway [201]. The HSE extract is a vasodilator, via its action on calcium channels, and it also exerts its effects by inhibiting the angiotensin converting enzyme (ACE) and by endothelial nitric oxide synthase (NOS) activation by the PI-3K/Akt pathway [202]. It is also a diuretic.

Our group has studied the effects of Hibiscus sabdariffa Linnaeus (HSL)-fed infusion on the OS in obese rats from a rat model of metabolic syndrome induced by high sucrose ingestion. The treatment with the HSL infusion decreased the lipoperoxidation and increased the total antioxidant capacity in the heart of MS rats and the activity of the enzymes Mn, Cu/Zn-SOD, peroxidases, GST activity. It also increased GHS, NO^3−^/NO^2−^ ratio. When these animals underwent ischemia/reperfusion, it restored the cardiac mechanical performance and coronary vascular resistance [203]. We also investigated the effects of Hibiscus sabdariffa Linnaeus (HSL)-fed infusion on the fatty acid (FA) profile in liver of metabolic syndrome (MS) rats. The treatment with the HSL decrease the disturbance of lipid metabolism in the liver, and it reduced FA and NEFAs [204].

#### 3.3.3. Garlic

Garlic has been consumed as a folk medicine all over the world for the prevention and treatment of many diseases, including obesity, since ancient times. It is a plant from the onion family (*Allium sativum* L.). Garlic effects are mainly due to the bioactive compounds it contains, which include sulfur compounds, such as organic sulfides, saponins, phenolic compounds, and polysaccharides [205]. Aged black garlic (ABG) is the source of aged and rusty garlic. ABG contains bioactive and organosulfuric compounds, such as diallyl thiosulfonate (allicin), E/Z-ajoene, S-allyl-cysteine (SAC), S-allyl-cysteine sulfoxide (alliin), β-resorcylic acid, pyrogallol, gallic acid, rutin, protocatechuic acid, quercetin, polysaccharides, fructose, glucose, galactose, flavonoids, phenols, thiosulfate, pyruvate, S-allylmercaptocysteine (SAMC), diallyl sulfide (DAS), diallyl disulfide (DADS), diallyl trisulfide (DATS), gamma-glutamyl tripeptides, sulfur dioxide (SO^2^), tetrahydro-beta-carboline derivatives, and diallyl tetrasulfide. These compounds of AGE are soluble in oil and are responsible for the antioxidant activity via activation of the Nrf2-ARE pathway [206,207]. The health benefits of garlic are caused by sulfur compounds, including diallyl disulfide and s-allyl cysteine. The sulfur compounds from garlic enter the body from the digestive tract and travel all over the body, where they exert their potent biological effects. Garlic contains antioxidants that support the body’s protective mechanisms against oxidative damage. High doses of garlic supplementation increase antioxidant enzymes in humans and reduce OS in subjects with high blood pressure [208].

In addition to the antioxidant effects of garlic, it also has anti-inflammatory effects. Among the ABG compounds that show anti-inflammatory effects are pyruvate, 5-hydroxymethylfurfural, and 2-linoleoylglycerol. Pyruvate can reduce bacteria lipopolysaccharide, which induces the increase of the inducible nitric oxide synthase (iNOS), and cyclooxygenase 2. In addition, 2-linoleoylglycerol suppresses the nitric oxide levels, prostaglandin E2, and pro-inflammatory cytokines via inhibition of mitogen activated protein kinases signaling pathways. The 5-hydroxymethylfurfural decrease adhesion monocytic cell in human umbilical vein endothelial cells incubated with TNF-α through the suppression of vascular cell adhesion molecule-1 expression, ROS generation, and NF-kB activation. ABG may also reduce insulin resistance, triglyceride levels, serum total cholesterol and increases the HDL levels. The possible mechanism action of garlic may be through down-regulation of the mRNA and the protein expression of PPARγ, C/EBPα, perilipin, adiponectin, plasminogen activator inhibitor 1, resistin, and TNF-α [209].

The risk of CVD, the anti-tumor and anti-microbial effects, and the reduction of high blood glucose concentration by garlic are caused by the different compounds it possesses. However, the exact mechanism of all ingredients and their long-term effects are not fully understood. Garlic also controls dyslipidemia by blocking cholesterol biosynthesis, decreasing lipid, and fibrinogen levels, diminishing the oxidation of LDL. A possible mechanism of action of garlic on dyslipidemia is by blocking the cholesterol biosynthesis enzymes, by reducing the absorption of cholesterol at the gut level, and by deactivating 3-hydroxy-3-methylglutaryl-CoA reductase, which participates in cholesterol biosynthesis. The substances of garlic that play a role in reducing lipid levels are allicin, SAC, and DADS. Allicin is a sulfur component of garlic that is formed from the interaction between the alliin enzyme and the substrate alliinase when the garlic is digested. It is a potent inhibitor in cholesterol synthesis [210].

Garlic also improves the insulin resistance underlies non-alcoholic fatty liver disease [211], through modulation of lipid metabolism and OS [212]. It also lowers blood pressure, blocks the progression of coronary artery disease, and elevates fibrinolytic activity. Some of the beneficial effects of garlic on cardiovascular disease and inflammation are related to the hydrogen sulfide (H_2_S) signaling pathway. H_2_S is endogenously produced by cystathionine synthetase (CBS), cystathionine lyase (CSE), and 3-mercaptopyruvate transferase (3-MST) under physiological conditions. DATS, the most potent polysulfide derived from garlic, significantly increased H_2_S. H_2_S as a lipophilic molecule, which is small and penetrates cell membranes without requiring transporters. It acts as a signaling molecule that controls important processes in the body. H_2_S interacts with many ion channels and receptors, as Ca^2+^, K_ATP_, Cl^−^ channels, TRVP1, and TRPA1 receptors, modulating different responses. In addition, it may regulate the Keap1-Nrf2 pathway, resulting in an increased expression of AREs. H_2_S release by garlic compounds requires the interaction with other low-molecular weight thiols, such as cysteine and GSH. Therefore, organic polysulfides derived from garlic are transported through the cell membrane and interact with GSH to generate H_2_S in red blood cells resulting in hyperpolarization in vascular smooth muscle cells and causing relaxation of the vessels [213].

Additionally, H_2_S acts as a gas transmitter modulating damage in ischemia/reperfusion, thus diminishing heart injury. It promotes the activity of adenosine triphosphate-sensitive potassium channels (K_ATP_) that affects several pro-inflammatory cytokines, and reduces H_2_O_2_, while elevating GSH levels.

H_2_S donors may also influence triglyceride levels via activation of the flux of autophagy in the liver, by blocking mTOR, which activates the autophagy pathway [214]. In this regard, H_2_S, can prevent the activation of the NF-kB signaling pathway, which consequently attenuates the production of pro-inflammatory cytokines. Other organosulfuric compounds in aged garlic extract, such as SAC, are also mediators of H_2_S by increasing its endogenous production, thus leading to the suppression of inflammation in obesity [206].

Our group has studied the antioxidant properties of aged garlic extract (AGE) in obese rats from a rat model of metabolic syndrome and on cardiovascular functioning. AGE returned levels of triglycerides, systolic blood pressure, insulin, leptin, HOMA index, and advanced glycation end products to their control values. AGE also reduced glutathion and GPx activity, and lipid peroxidation. There is increased vasocontraction and reduced vasodilation in rats from a metabolic syndrome model, and AGE diminished it. Coronary vascular resistance was increased in MS rats, and AGE decreased it. Thus, AGE diminished MS-induced cardiovascular risk, through its antioxidant properties [215].

## 4. Summary and Conclusions

In summary, OS plays an important role in the pathophysiology of obesity, altering the function of regulatory factors of mitochondrial activity, modifying the concentration of molecules taking part in inflammation, which is associated with a large number and size of adipocytes, promoting lipogenesis, stimulating the differentiation of preadipocytes to mature adipocytes adipogenesis and playing an important role as agents that regulate the energy balance in hypothalamic neurons that control appetite. Therefore, the natural antioxidants (natural compounds found in many plants) play important roles controlling obesity. Important groups of compounds found in plants with antioxidant properties include (a) polyphenols, including phenolic acids, stilbenes, flavonoids (flavonols, flavanols, anthocyanins, flavanones, flavones, flavanonols, and isoflavones), chalcones, lignans, and curcuminoids (b) carotenoids, (c) capsaicinoids and casinoids, (d) isothiocyanates, and (e) catechins. Examples of these compounds are resveratrol, quercetin, curcumin, ferulic acid, phloretin, components found in green tea such (-)-epicatechin, (-)-epicatechin-3-gallate, (-)-epigallocatechin, and (-)-epigallocatechin-3-gallate (EGCG), and components of Hibiscus Sabdariffa extracts that include anthocyanins, delphinidin, hibiscetin, quercetin, and gossypetin), protocatechuic acid alkaloids, and L-ascorbic acid, among others. The antioxidant activities depend on their scavenging of ROS activities and the prevention of NF-κB activation, with subsequent reduction of the expression of target genes. Therefore, natural compounds may have therapeutic potential for diseases mediated by oxidative stress, particularly obesity. Controlled and well-designed clinical trials are still necessary to better know the effects of these compounds.

## Data Availability

Data sharing not applicable.
